# Procalcitonin and C-reactive protein perform better than the neutrophil/lymphocyte count ratio in evaluating hospital acquired pneumonia

**DOI:** 10.1186/s12890-020-01207-6

**Published:** 2020-06-11

**Authors:** Nan Zheng, Dongmei Zhu, Yi Han

**Affiliations:** Department of Critical Care Medicine, the First Affiliated Hospital of Nanjing Medical University, Nanjing Medical University, 300 Guangzhou Road, Nanjing, 210029 China

**Keywords:** Hospital-acquired pneumonia, Neutrophil/lymphocyte count ratio, Procalcitonin, C-reactive protein, Composite biomarker

## Abstract

**Background:**

The relationship between biomarkers and hospital-acquired pneumonia (HAP) is understudied, especially in severe cases admitted to the intensive care unit (ICU). Compared with community-acquired pneumonia (CAP), HAP might have different traits regarding biomarkers due to the previous history in hospitals.

**Methods:**

A total of 593 adult patients were enrolled in this retrospective cohort study to determine the neutrophil/lymphocyte count ratio (NLCR), procalcitonin (PCT), C-reactive protein (CRP) and serum lactate level upon admission to the ICU. According to diagnosis, patients were divided into two groups: non-infection and HAP. Discriminant analysis was performed based on better outcomes of diagnostic performance and severity evaluation. The diagnostic performance of each individual biomarker was assessed by constructing receiver operating characteristic (ROC) curves and calculating the area under each ROC curve (AUROC). Multivariable analysis was also applied to determine the most appropriate prognostic factors.

**Results:**

NLCR, PCT and CRP were markedly different between the non-infection and HAP groups. NLCR had a worse ability to discriminate severe infection (AUROC 0.626; 95% CI 0.581–0.671) than conventional markers such as CRP (0.685, 95% CI 0.641–0.730) and PCT (0.661, 95% CI 0.615–0.707). In addition, the AUROC of composite biomarkers, especially the combination of NLCR, CRP and WBC, was significantly greater than that of any single biomarker.

**Conclusions:**

NLCR was not comparable to conventional single biomarkers, such as CRP and PCT, for diagnosing or evaluating the severity of HAP. Composite biomarkers that have good accessibility, especially the combination of NLCR, CRP and WBC, could help with early diagnosis and severity evaluation.

## Background

Hospital-acquired pneumonia (HAP) is pneumonia acquired during hospitalization in patients with or without invasive mechanical ventilation. HAP is frequent in critical patients and remains the leading cause of death among hospital-acquired infections [1]. This life-threatening conditions of patients in the intensive care unit (ICU) may require mechanical ventilation associated with prolonged hospital stay and high mortality. Certain clinical and laboratory parameters have been applied to facilitate the diagnosis, evaluate the severity, guide antibiotic administration for and predict the prognosis of HAP. However, most of them have not proven effective enough for severity evaluation and outcome prediction.

Procalcitonin (PCT) is a useful serum marker in the prediction, diagnosis and severity evaluation of bacterial infections in critically ill patients [2]. It has been shown to be associated with the severity of inflammation and prognosis during sepsis and septic shock [3, 4]. Some large studies have demonstrated that increased levels of PCT were associated with a bacterial etiology of community acquired pneumonia (CAP) and adverse short-term outcome [5]. The usefulness of PCT has emphasized that different-level patterns may be useful to guide antimicrobial therapy in patients with various infections, such as community-acquired lower respiratory tract infection [6], ventilation-acquired pneumonia [7], blood stream infection [8] and abdominal infection [9].

C-reactive protein (CRP), a highly conserved plasma protein, is a homopentameric acute-phase inflammatory protein that was initially discovered in 1930 by Tillet and Francis. CRP exhibits elevated expression during inflammatory conditions such as rheumatoid arthritis, cardiovascular disease and infection. As an acute-phase protein, the plasma concentration of CRP deviates by at least 25% during inflammatory disorders [10]. CRP, as a conventional biomarker, also plays a key role in identifying and evaluating bacterial infections [11]. Although it has proven predictive of the severity of pneumonia, very few, poor-quality investigations have compared its potential with NLCR for the evaluation of inflammation [12].

Numerous studies have evaluated the diagnostic and evaluative performance of the neutrophil/lymphocyte count ratio (NLCR) in various clinical conditions, such as sepsis [13], septic shock [14], bacteremia [15, 16], renal, lung and colorectal carcinomas and intracranial tumors [17]. In addition, NLCR can predict the severity and outcome of CAP with higher prognostic accuracy than traditional infection markers in the emergency department [12].

Composite biomarkers have been applied to evaluate respiratory tract infection, but few studies have focused on their diagnostic accuracy and prognostic utility. Some findings have shown greater value for composite biomarkers in discriminating the severity of inflammation [18–20]. In this study, we compared the diagnostic performance of composite biomarkers with single biomarkers.

As mentioned above, different biomarkers have been applied to evaluate the severity of inflammation. On their diagnostic accuracy and predictive value, there are diverse opinions. Currently, it is still controversial whether NLCR may be less suitable to detect the presence of sepsis in ICU patients [13]. Moreover, data comparing NLCR and conventional markers in patients with HAP are very limited. Thus, this study aimed to clarify whether NLCR presents advantages over conventional markers or whether composite biomarkers could be a better choice for the diagnosis and evaluation of HAP.

## Methods

### Patients and study design

This retrospective study was conducted with data collected from Jan 2017 to June 2019 at the First Affiliated Hospital of Nanjing Medical University, a tertiary hospital with more than 2000 beds, in the southeast region of China. Patients admitted to the ICU on suspicion of HAP or without infection were consecutively enrolled in this study. Patients with other infections, such as cholecyst, skin and soft tissue, urinary system, abdomen or central nervous system infections, were excluded. All physiological and pathophysiological data and laboratory and microbiological results were recorded. For microbiological analysis, culture findings were based upon both airway samples and blood culture. PCR (polymerase chain reaction)- and EIA (enzyme immunoassay)-based methods were used for virus and antibody detection. The outcome in this study was 28-day survival, which was recorded according to both hospital mortality and proper follow-up.

Each medical record was subjectively reviewed by two senior specialists in ID. According to the HAP definition, these two independent specialists in infectious disease/critical care medicine followed the standard principles to recruit the cohort. Uncertainties were ruled out according to clinical symptoms, blood tests, microbiological tests and radiographic imaging.

HAP was defined in patients who developed pneumonia after 48 h of hospitalization when not receiving invasive mechanical ventilation (iMV) [21, 22]. We recruited HAP patients at admission. Some of the patients required mechanical ventilation or noninvasive ventilatory support after admission. The clinical diagnosis of pneumonia was based on clinical criteria as suggested in guidelines [21, 23, 24]: (1) new or progressive radiologic pulmonary infiltrate together with (2) at least two of the following: temperature > 38 °C or < 36 °C, leukocytosis > 12,000/mm3 or leukopenia < 4000/mm3, and purulent respiratory secretions.

#### Inclusion criteria

(1) age 18 to 89 years; (2) admission to the ICU of the First Affiliated Hospital of Nanjing Medical University during the period from Jan 2017 to Jun 2019; and (3) diagnosis of HAP or non-infectious disease.

#### Exclusion criteria

(1) hematological disease; (2) chemotherapy; (3) receiving glucocorticoids; and (4) receiving bone marrow stimulators.

Enrolled patients were divided into two groups according to the diagnosis: (1) non-infection group: infection of any origin and by any organism was ruled out; (2) HAP group: the HAP criteria were met.

### Statistical analysis

All continuous variables are expressed as the median and interquartile range because they were not normally distributed. The F test was used to compare variances of continuous variables between two groups. If variances were significantly different, the unpaired t test with Welch’s correction was applied. If variances were equal, the Mann-Whitney U test was applied. *p* < 0.05 was considered significant.

Composite biomarkers were constructed using bivariate logistic regression analysis. The different composites consisted of PCT, CRP and/or NLCR. The most valuable composite was chosen, i.e., the one that presented the highest discriminant capability between groups. A comparison of the diagnostic accuracy of the biomarkers, alone and in combination, was made by receiver operating characteristic (ROC) curve analysis by calculating the area under the curve (AUROC). For comparison of AUROCs, the Mann-Whitney U test for two correlated ROC curves was used. All tests were two-sided, and *p* < 0.05 was considered statistically significant. The statistical analysis and graph construction were performed using SPSS 23 and Stata 12.

## Results

### General characteristics

**Fig. 1:**
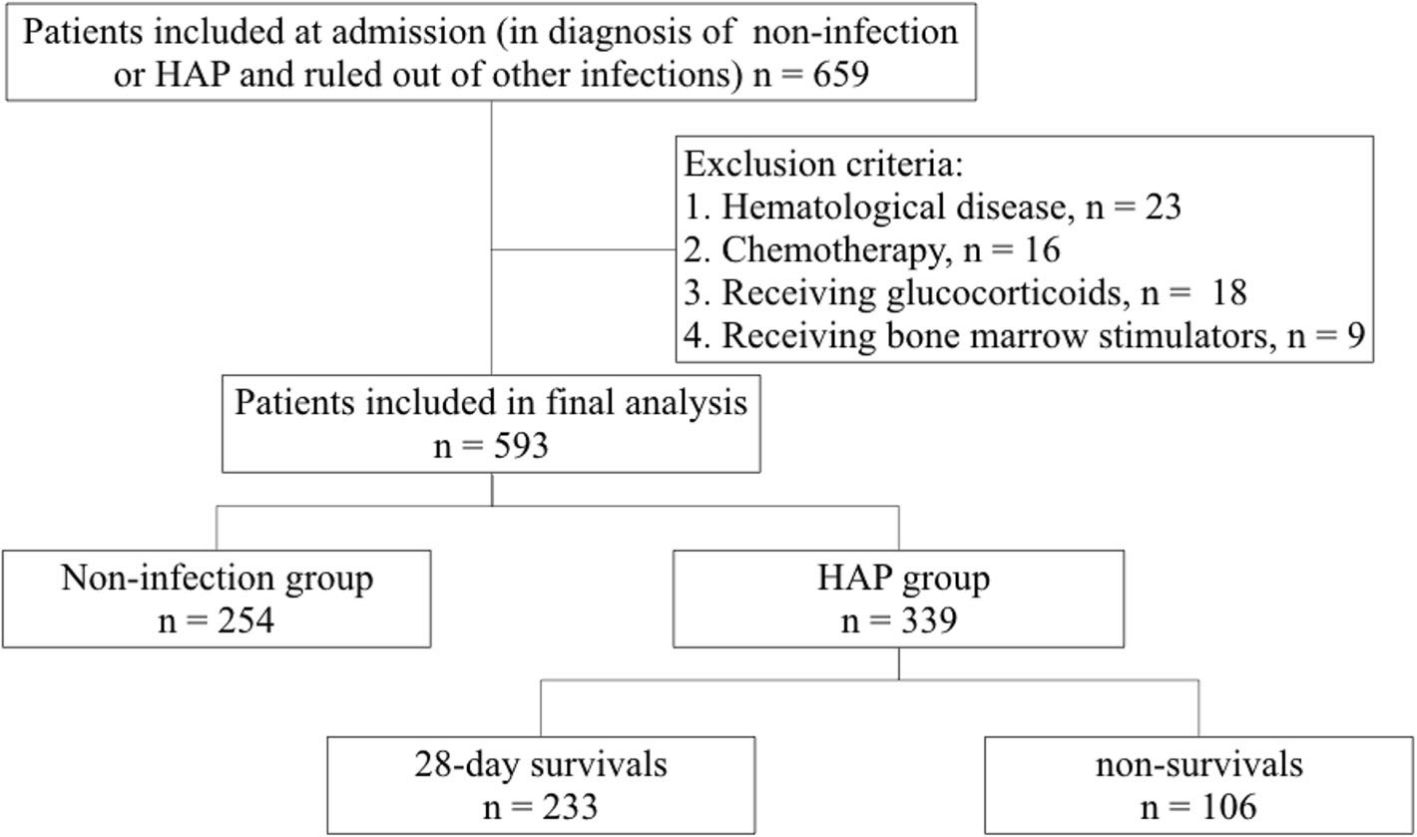
Enrollment flowchart. Patients in diagnosis of non-infection or HAP were included at admission. Patients with other infection, such as infection of cholecyst, skin and soft tissue, urinary system, abdomen or central nervous system were all excluded

A total of 659 episodes in adult patients suspected of having HAP or no infection admitted to the ICU at the First Affiliated Hospital of Nanjing Medical University were enrolled. Among the total enrolled population, 66 patients were excluded from the analysis because they met one or more exclusion criteria (Fig. 1).

General characteristics of the overall population are displayed in Table 1. As a severity score for evaluation of the disease, APACHE II in the HAP group was significantly higher than in the non-infection group (Table 1). Apart from this, most biomarkers, including NLCR, PCT, CRP and WBC, in the HAP group were significantly higher than in the non-infection group, as described in Table 1.

**Table 1:** Characteristics of the overall population

	Non-infection ***n*** = 254	HAP ***n*** = 339
**Age (years)**	66.6 ± 17.1	71.0 ± 17.9 **
**Sex (M / F)**	152 / 102	252 / 87 *
**WBC abnormalities, n, (%)**	54 (21.3%)	130 (38.2%) *
**NE abnormalities, n, (%)**	183 (72.0%)	254 (74.7%) *
**APACHE II score (mean ± sd)**	17.7 ± 5.9	21.7 ± 6.1 ***
**NLCR**	10.2 ± 9.8	13.0 ± 17.1 *
**PCT (ng/ml)**	0.75 ± 3.25	4.60 ± 15.97 ***
**CRP (mg/ml)**	45.8 ± 50.6	73.3 ± 79.6 ***
**Blood lactate (mmol/l)**	1.6 ± 1.3	1.5 ± 0.8
**Surgery, n, (%)**	100 (39.4%)	87 (25.6%) **
**28 days survival, n, (%)**	205 (80.7%)	233 (68.5%) **
**Diabetes mellitus, n, (%)**	5 (2.0%)	0 (0) *
**Cardiovascular disease, n, (%)**	28 (11.0%)	18 (5.3%) *
**Hypertension, n, (%)**	5 (2.0%)	1 (0.3%) *
**Malignancies, n, (%)**	54 (21.3%)	10 (2.9%) ***
**COPD, n, (%)**	10 (3.9%)	17 (5.0%)
**Liver cirrhosis, n, (%)**	2 (0.8%)	0 (0)
**Renal failure, n, (%)**	2 (0.8%)	16 (4.7%) **

Data were expressed as Mean ± standard deviation or number (percentage) of current group. *p < 0.05, **p < 0.01, ***p < 0.001 vs non-infection group. *WBC* white blood count; *NE* neutrophil; *NLCR* neutrophil/lymphocyte count ratio; *PCT* procalcitonin; *CRP* C-reactive protein; *HAP* hospital acquired pneumonia; *COPD* chronic obstructive pulmonary disease

The incidence of cardiovascular comorbid conditions on admission to the ICU was lower in patients with non-infection than in the HAP group. On the other hand, the incidence of malignancies was much higher in the non-infection group. The comorbid disease profile is presented in Table 1. Additionally, the surgery incidence in the non-infection group was much higher than in the HAP group (Table 1).

### Microorganism profile

Positive cultures of the microbiological samples taken within 48 h of admission were reported in all episodes of HAP patients, and these totaled 324 cultures with microorganisms. Among all analyzed cases, 237 bacterial isolates were found in 237 episodes, with 27 isolates of gram-positive organisms and 210 isolates of gram-negative organisms. Apart from these, 83 isolates of fungi and 3 of viruses or antibodies were identified. The detected microorganism profile is shown in Table 2.

**Table 2:** Microorganism profile for the patients in the study cohort

	28-day survivors ***n*** = 235	28-day non-survivors ***n*** = 104	***P*** value
**Gram-positive isolates (n, %)**	**16 (6.8%)**	**11 (10.6%)**	**0.2772**
S.Aureus	12 (5.1%)	10 (9.6%)	0.1505
MRSA	2 (0.9%)	0 (0)	1
Streptococcus spp.	1 (0.4%)	0 (0)	1
Enterococcus spp.	2 (0.9%)	1 (1.0%)	1
Other	1 (0.4%)	0 (%)	1
**Gram-negative isolates (n, %)**	**158 (67.2%)**	**52 (50.0%)**	**0.0035**
Acinetobacter baumannii	76 (32.3%)	37 (35.6%)	0.6175
Klebsiella spp.	59 (25.1%)	26 (25.0%)	1
Pseudomonas spp.	50 (21.3%)	13 (12.5%)	0.0687
Enterobacter spp.	24 (10.2%)	6 (5.8%)	0.2177
*S. maltophilia*	16 (6.8%)	3 (2.9%)	0.2018
Other	9 (3.8%)	4 (3.8%)	1
**Fungi isolates (n, %)**	**55 (23.4%)**	**28 (26.9%)**	**0.4959**
*Candida albicans*	28 (11.9%)	17 (16.3%)	0.2985
Candida glabrada	16 (6.8%)	2 (1.9%)	0.0702
*Candida tropicalis*	7 (3.0%)	4 (3.8%)	0.7423
Other	4 (1.7%)	4 (3.8%)	0.2556
**Virus isolates (n, %)**	**2 (0.9%)**	**1 (1.0%)**	1
**Tuberculosis isolates (n, %)**	**1 (0.4%)**	**0 (0)**	1

Data were presented as number of isolates (percentage of current group), not number of patients. *S. aureus*, *Staphylococcus aureus*; MRSA, methicillin-resistant *Staphylococcus aureus*; S. maltophilia, Stenotrophomonas maltophilia; spp., species

### Diagnostic performance of the markers

**Fig. 2:**
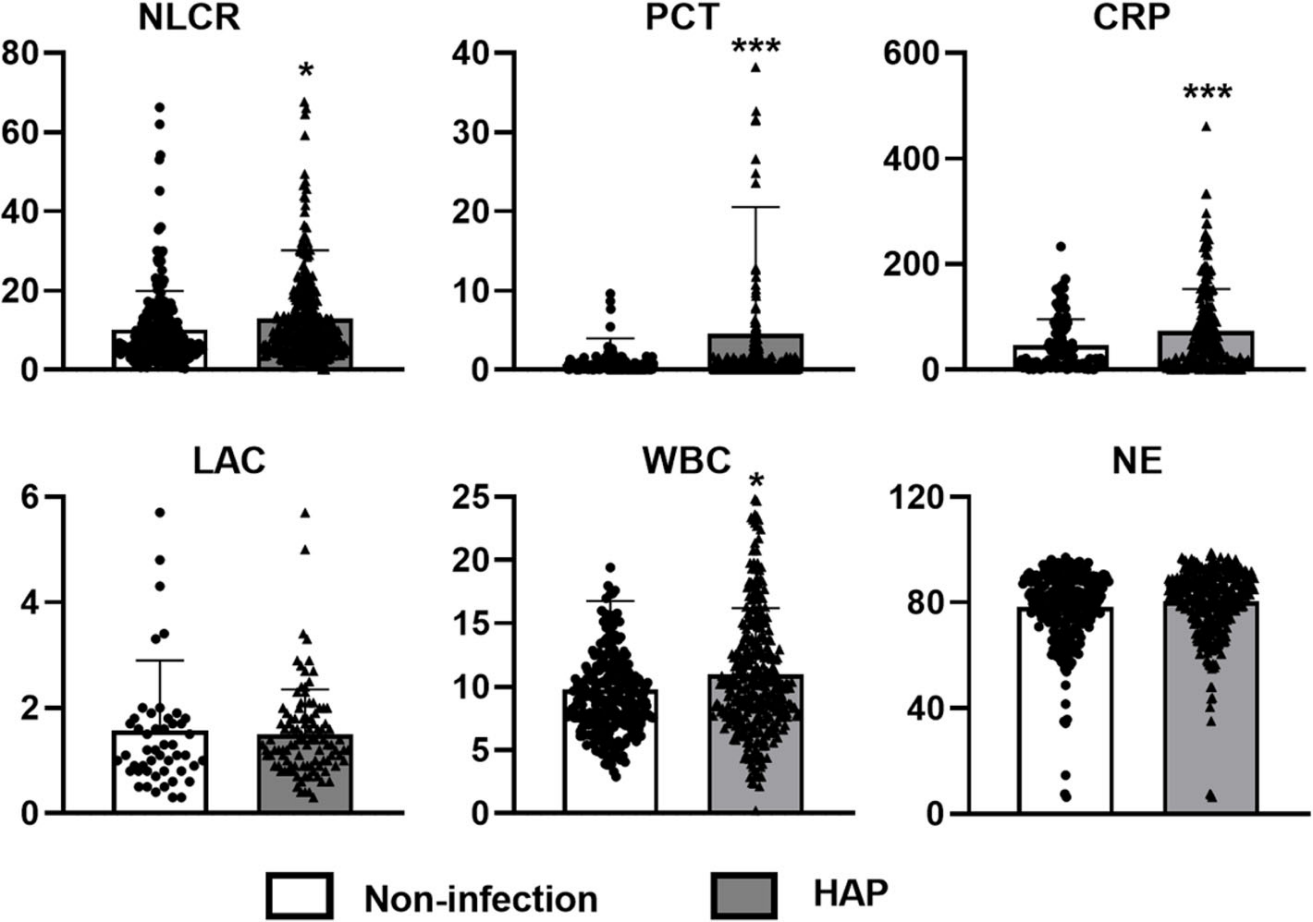
Single biomarker levels of NLCR, PCT, CRP, LAC, WBC, and NE in non-infection and HAP group. *p < 0.05, ***p < 0.001 vs non-infection group. NLCR: neutrophil/lymphocyte count ratio; PCT: procalcitonin; CRP, C-reactive protein; LAC: lactate; WBC: white blood count; NE: neutrophil %; HAP, hospital acquired pneumonia

Serum levels of various biomarkers between different groups were compared to determine the discriminant capability. Our study showed that PCT, CRP, NLCR and WBC were all distinguishable between the two groups. However, serum lactate (LAC) and neutrophil % (NE) did not present any difference between these two groups. Compared to NLCR, both PCT and CRP levels showed good differential ability between the non-infection and HAP groups (Fig. 2).

**Fig. 3:**
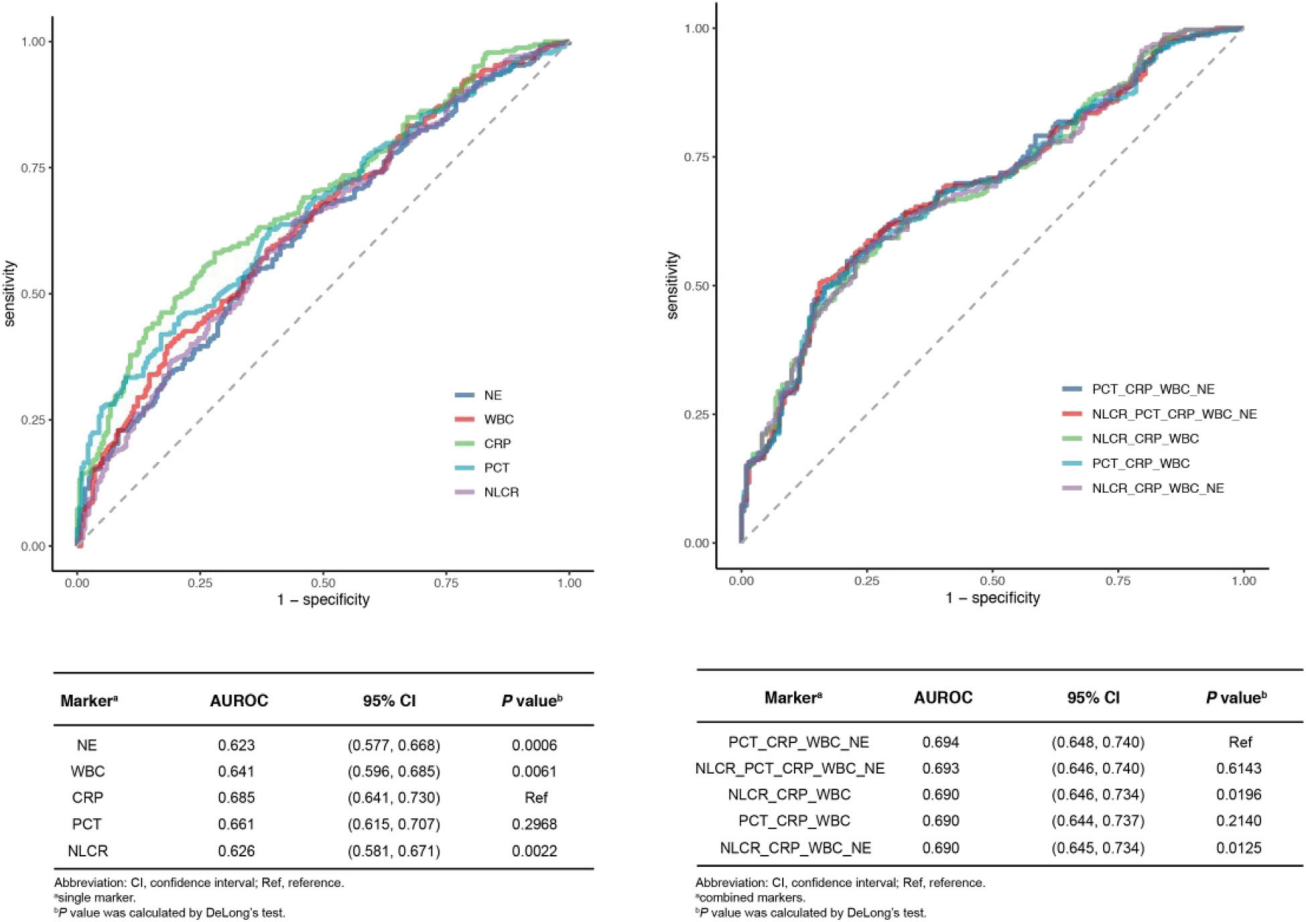
Receiver operator characteristic (ROC) curve for HAP discrimination with non-infection group, and area under the ROC (AUROC) for the single and top five combined biomarkers evaluated in this study. NE: neutrophil%; WBC: white blood count; CRP, C-reactive protein; PCT: procalcitonin; NLCR: neutrophil/lymphocyte count ratio.

In the ROC curve analysis, the single biomarkers CRP (AUC 0.685; 95% CI 0.641–0.730) and PCT (AUC 0.661; 95% CI 0.615–0.707) presented a greater ability to differentiate HAP patients from non-infected patients than NLCR (AUC 0.626; 95% CI 0.581–0.671), WBC (AUC 0.641; 95% CI 0.596–0.685) or NE (AUC 0.623; 95% CI 0.577–0.668). Compared to single biomarkers, combined markers showed slightly better discriminant ability and diagnostic performance between the two groups (Fig. 3).

As analyzed above, LAC was similar between verified HAP patients and non-infected patients, based on the median (interquartile) analysis (Table 1 and Fig. 2) and AUROC calculation.

We tested all the possible composites of single biomarkers and display the top 5 AUROCs of composite biomarkers in Fig. 3. The results showed that the top five combinations had similar AUROCS (Fig. 3). For the purpose of easy accessibility, the combinations of NLCR-CRP-WBC (AUC 0.690; 95% CI 0.646–0.734) and PCT-CRP-WBC (AUC 0.690; 95% CI 0.644–0.737) were deemed the most valuable in this study, as other combinations involved four or five single biomarkers (Fig. 3).

### Survival and mortality

The overall 28-day mortality rate of the enrolled population was 25.3% (*n* = 150), and it was 30.7% (*n* = 104) in the HAP group and 18.1% (*n* = 46) in the non-infection group. The mortality rate in the HAP group was significantly higher than that of the non-infection group.

**Fig. 4:**
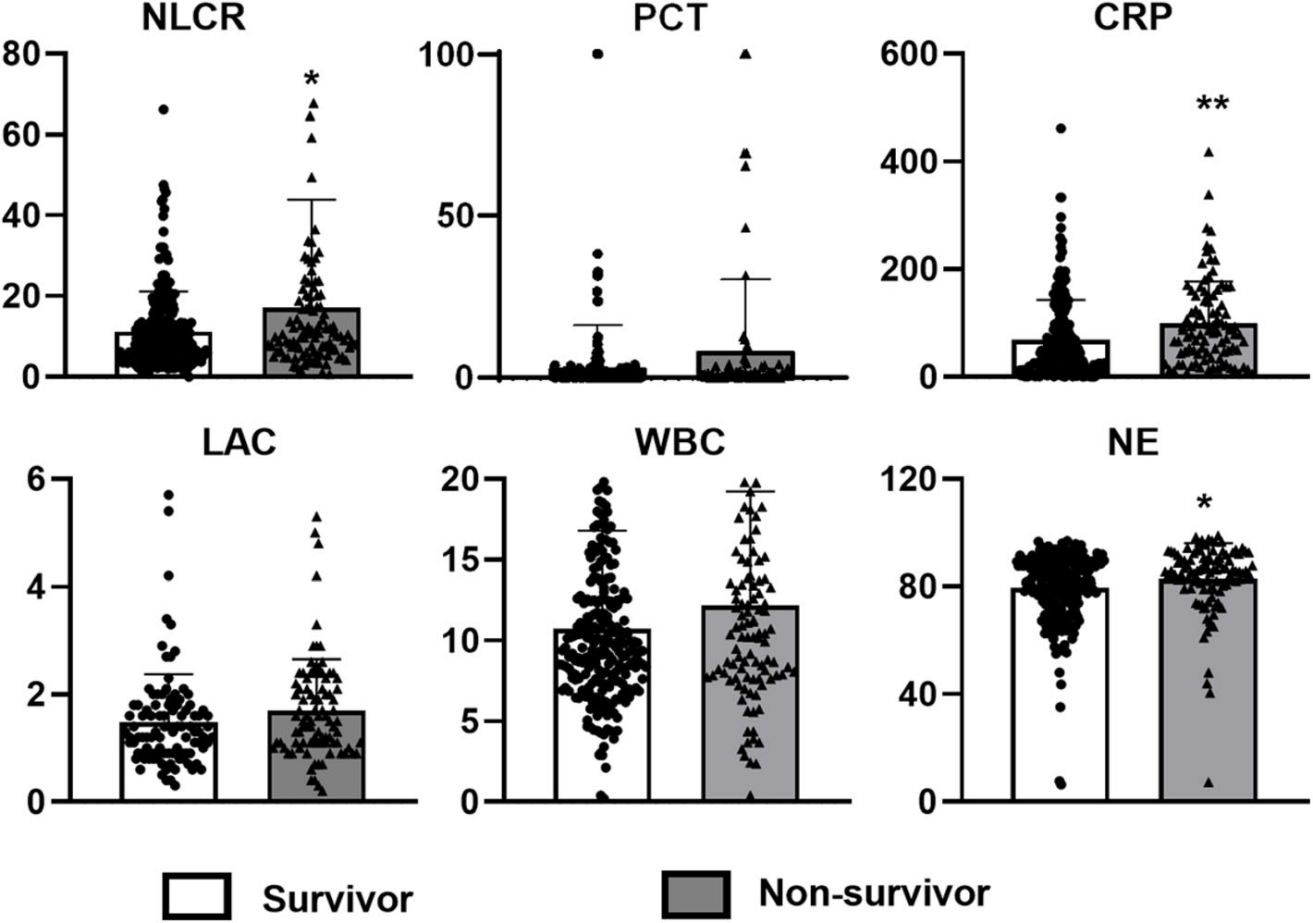
Single biomarker levels of NLCR, PCT, CRP, LAC, WBC and NE in 28-d survivals and non-survivals of HAP group. *p < 0.05, **p < 0.01 vs 28-d survivals. NLCR: neutrophil lymphocyte count ratio; PCT: procalcitonin; CRP, C-reactive protein; LAC: lactate; WBC: white blood count; NE: neutrophil %

**Fig. 5:**
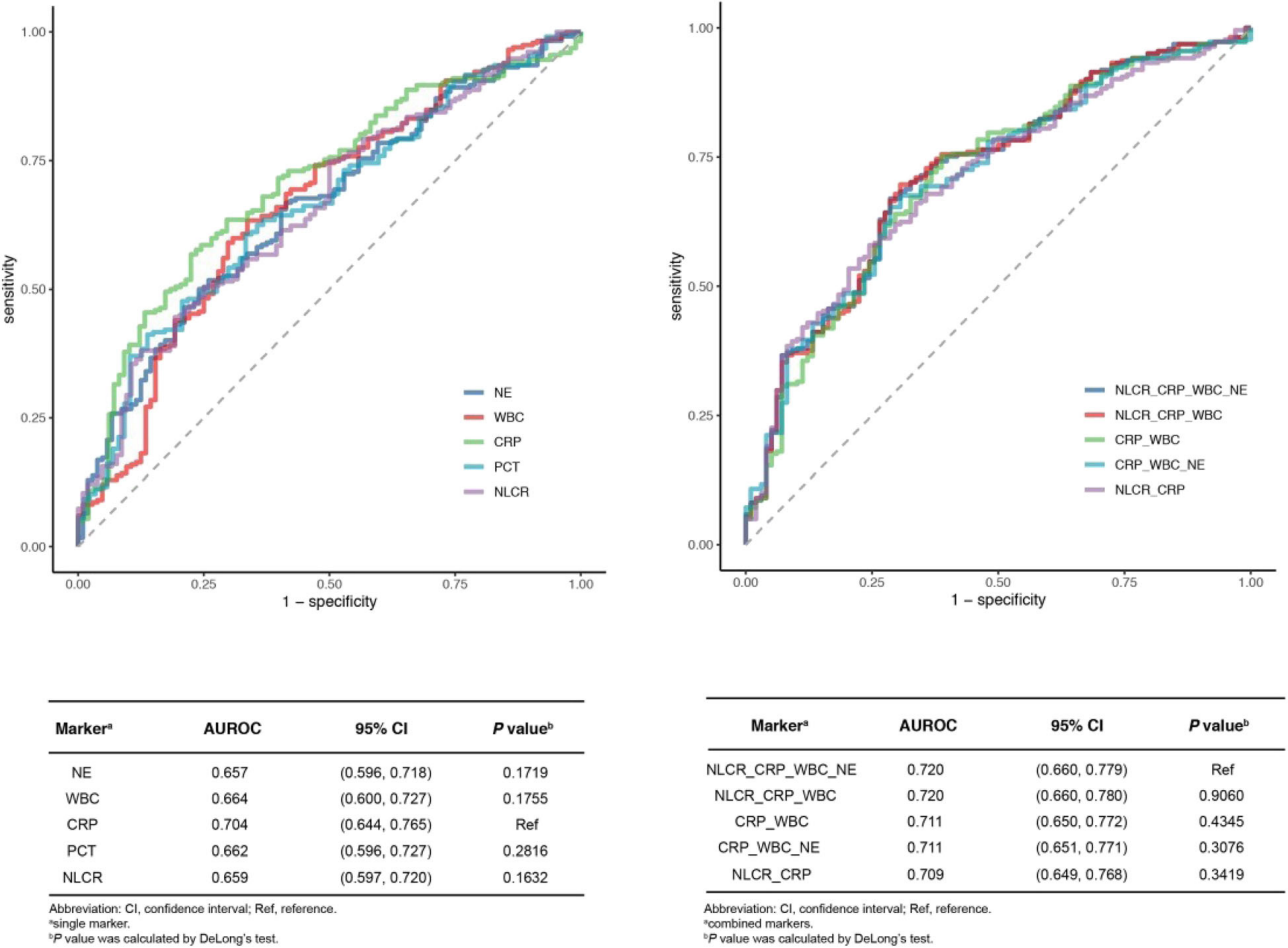
Receiver operator characteristic (ROC) curve for 28-day survival discrimination with non-survival in HAP group and area under ROC (AUROC) for the single and top five combined biomarkers evaluated in HAP group. NE: neutrophil %; WBC: white blood count; CRP, C-reactive protein; PCT: procalcitonin; NLCR: neutrophil/lymphocyte count ratio

In the statistical analysis of 28-day survival in the HAP group, NLCR, CRP and NE were much higher in the nonsurviving population, indicating that all these markers might have the potential to predict the prognosis of HAP patients (Fig. 4). The ROC analysis showed that CRP (AUC 0.704; 95% CI 0.644–0.765) had the greatest discriminant ability to predict 28-day mortality as a single marker. Other single markers, such NLCR, PCT, WBC and NE, also showed good predictive value for outcomes (Fig. 5).

In regard to composite biomarkers, the composite of NLCR-CRP-WBC presented to be the most valuable to predict mortality of HAP patients, as its AUROC (AUC 0.720; 95% CI 0.660–0.780) was the best among all compositions (Fig. 5). Other combinations of biomarkers, such as NLCR-CRP (AUC 0.709; 95% CI 0.649–0.768) and CRP-WBC (AUC 0.711; 95% CI 0.650–0.772), were also as potent as NLCR-CRP-WBC but with easier accessibility and simpler combinations. Compared to most single biomarkers, combined markers showed better discriminant ability and diagnostic performance between the survivors and nonsurvivors. We tested all the possible compositions of single biomarkers and display the top 5 AUROCs of composite biomarkers in Fig. 5.

## Discussion

Over the past few years, numerous studies have investigated the clinical value of various biomarkers in the diagnosis, prognosis and stratification of pneumonia [25]. Many studies have focused on the significance of single biomarkers, but interest in multiple biomarkers has increased, especially in the severity evaluation of infective conditions, such as sepsis, septic shock and CAP. Contradictory conclusions have been presented due to inconsistent results drawn from small samples [26, 27]. In this study, we investigated the clinical value of NLCR, PCT and CRP alone and in combination in a large sample (593 episodes in adult patients).

Here, we found that patients with HAP had higher levels of PCT and CRP than patients without HAP (Table 1 and Fig. 2). These patients showed greater severity as shown by higher APACHE II scores (Table 1). This is in agreement with the data from Liu et al., who reported that PCT was associated with the severity of illness in patients with severe pneumonia and appeared to be a prognostic marker of morbidity and mortality comparable to the APACHE II score [27]. Our results suggested that CRP performed best in discriminating HAP with non-infection status as a single indicator (Figs. 2,3). This agrees with the previous findings that PCT was a valuable marker to predict mortality in septic patients [28].

PCT and CRP, as traditional biomarkers, have been intensely investigated for the past few years, and the outcomes have been contradictory [27, 29]. This may be caused by the small sample size, which could undermine the overall reliability of the results [30]. Both CRP and PCT can be affected by severe inflammatory status, even of non-infectious origin. This might partially explain why the AUROCs of these biomarkers were relatively low. In addition, any biomarker could be used if incorporating clinical judgment. In our present study, PCT demonstrated a relatively high differential value (Figs. 2, 3) between HAP and non-infection patients, indicating that the patients with a higher level of PCT could be likely diagnosed with HAP, in combination with a clinical history. This is in line with the study of Heggelund L that found that inflammatory biomarkers were associated with etiology and predicted outcomes in community-acquired pneumonia [5].

NLCR has been reported to be an indicator that correlates with the severity of a series of diseases and has been applied to predict the prognosis of various clinical circumstances, ranging from colorectal cancer [17], glial tumor [31], sepsis and/or septic shock [14, 18] to acute coronary syndrome [32]. In this study, we compared NLCR with other biomarkers on diagnostic performance and prognostic prediction. The conclusion was that NLCR could be a valuable marker to help with the diagnosis of HAP and the prediction of mortality. On the other hand, conventional markers such as CRP and PCT both performed better than NLCR in discriminating HAP and non-infected patients in this investigation, as shown in Fig. 3. NLCR did perform similarly to both CRP and PCT in the prediction of outcomes, as described in Fig. 5.

Regarding the AUROC analysis, CRP performed the best as a single biomarker to discriminate HAP from non-infected patients and to predict 28-day mortality in the HAP group (Figs. 3, 5). Meanwhile, PCT showed good value for discriminating HAP from non-infected patients and for predicting 28-day mortality in the HAP group (Figs. 3, 5). On the other hand, NLCR presented good diagnostic performance and outcome prediction ability, and only its value on diagnostic performance was weaker than that of CRP. We compared our results with previous literature [18, 19] and found that many factors that affect the diagnostic performance of a biomarker might have cause controversy we have seen, thereby making it difficult and less viable to compare results from different studies.

In this study, we employed different methods to combine several biomarkers into one variable. Taking accessibility into account, the composition of NLCR-CRP-WBC had the most valuable diagnostic and prognostic performance out of all the combinations, based on our AUROC analysis. Thus, we deemed this combination the best composite. The composite biomarker NLCR-CRP-WBC proved to be a reasonable predictor of 28-day mortality, indicating that it might also be employed for risk stratification purposes. This 3-marker composite may be applied to HAP patients for severity evaluation. This composite biomarker presented a significantly higher AUROC than most single biomarkers, suggesting that the joint interpretation of multiple biomarkers could be more valuable in the evaluation of HAP.

With respect to the 28-day survival analysis, the survival rate of the HAP group was much lower than that of the non-infection group. The majority of the biomarkers in nonsurvivors were significantly elevated. These data agree with previous observations that PCT in pneumonia patients correlated with the risk of death independent of the clinical risk assessment [33, 34]. In addition, PCT can identify unfavorable outcomes in CAP and VAP (ventilator acquired pneumonia) patients in the ICU [35–37]. In clinical scenarios, PCT is more frequently used to guide antibiotic treatments. Although many reports support NLCR as a valuable marker for the severity evaluation and prognosis prediction of infectious diseases [14–16], our study puts its value into question. Compared with conventional biomarkers such as CRP and PCT, NLCR presented moderate values in HAP diagnostic and prognostic performance.

## Limitations of the study

Several limitations of this study could cause concern. First, patients with antibiotic treatment were not excluded from this study. Therefore, false-negative results may be generated, leading to underestimation of the severity of disease. Second, the clinical diagnosis of HAP may lack accuracy in some cases, where there were no consistent changes on chest imaging, or there might be false-negative results of microbiological sampling in patients receiving broad-spectrum antibiotics for a clinical diagnosis of HAP, or there might be positive results of microorganisms to be diagnosed as HAP, only in fact due to certain inflammation status combined with hospital-acquired bacterial colonization instead of bacterial infection. Third, in the non-infection group, this was true only for the admission period because these patients could have gotten an infection afterwards. In this study, we only evaluated the data of patients at admission. Different infections are caused by various stimuli. Due to the totally different mechanisms underlying the processes of infection, our study focuses on HAP and looked for sensitivity of biomarkers for HAP. More studies should be performed to detect the sensitivity of biomarkers of infected patients suffering from any other severe infections, such as infections of the cholecyst, skin and soft tissue, urinary system, abdomen or central nervous system.

## Conclusions

In patients with HAP, conventional biomarkers such as CRP and PCT were associated with the severity of the disease and could be good prognostic markers for the prediction of morbidity and mortality in these patients. NLCR, as a recently explored biomarker, presented no advantage over conventional markers on severity evaluation or prognostic prediction of HAP. Both CRP and PCT performed better than NLCR in the severity evaluation of HAP. Multiple-biomarker composites could be a better choice for the purpose of disease diagnosis, severity evaluation, treatment guidance and prognostic prediction for HAP patients, especially the NLCR-CRP-WBC composite, as it is characterized by easy access, simple interpretation and reliable quality.

## Data Availability

The datasets used and/or analyzed during the current study are available from the corresponding author on reasonable request.

## Abbreviations

APACHE II: Acute Physiology and Chronic Health Evaluation II
AUROC: Area under the ROC curve
CAP: Community acquired pneumonia
CI: Confidence interval
CRP: C-Reactive protein
EIA: Enzyme immunoassay
HAP: Hospital acquired pneumonia
iMV: Invasive mechanical ventilation
IRB: Institutional review boards
LAC: Lactate
NE: Neutrophil %
NLCR: Neutrophil/lymphocyte count ratio
PCT: Procalcitonin
PCR: Polymerase chain reaction
ROC: Receiver operating characteristic
WBC: White blood cell
